# First Detailed Genetic Characterization of the Structural Organization of Type III Arginine Catabolic Mobile Elements Harbored by Staphylococcus epidermidis by Using Whole-Genome Sequencing

**DOI:** 10.1128/AAC.01216-17

**Published:** 2017-09-22

**Authors:** Brenda A. McManus, Aoife M. O'Connor, Peter M. Kinnevey, Michael O'Sullivan, Ioannis Polyzois, David C. Coleman

**Affiliations:** a Microbiology Research Unit, Division of Oral Biosciences, Dublin Dental University Hospital, Trinity College Dublin, University of Dublin, Dublin, Republic of Ireland; b Division of Restorative Dentistry and Periodontology, Dublin Dental University Hospital, Trinity College Dublin, University of Dublin, Dublin, Republic of Ireland

**Keywords:** ACME, Staphylococcus epidermidis, oral cavity, ST329

## Abstract

The type III arginine catabolic mobile element (ACME) was detected in three Staphylococcus epidermidis oral isolates recovered from separate patients (one healthy, one healthy with dental implants, and one with periodontal disease) based on ACME-*arc*-operon- and ACME-*opp3*-operon-directed PCR. These isolates were subjected to whole-genome sequencing to characterize the precise structural organization of ACME III for the first time, which also revealed that all three isolates were the same sequence type, ST329.

## TEXT

The arginine catabolic mobile element (ACME) was first described in the Staphylococcus aureus strain USA300 (1) and is thought to aid colonization and persistence on skin. Since it was first described, ACMEs ranging from 30 to 34 kb in size have been identified in other staphylococcal species, including Staphylococcus epidermidis (1, 2–5). The element is primarily characterized by the presence of two distinct operons: the *arc* operon (*arcR/A/D/B/C*), which encodes an arginine deaminase pathway, and the *opp3* operon (*opp3A/B/C/D/E*), which encodes an oligopeptide permease ABC transporter. To date, three distinct types of ACME have been described based on (i) the presence of both *arc* and *opp3* operons (type I), (ii) the *arc* operon only (type II), and (iii) the *opp3* operon only (type III). The genetic structure and organization of ACME types I and II in staphylococci have been elucidated in detail previously, including by the use of whole-genome sequencing (WGS) (1, 4). In contrast, the corresponding genetic structural organization of ACME type IIIs have not been comprehensively characterized to date. What is known about ACME IIIs in staphylococci is based on PCR-based scanning/tiling methods using primer pairs designed against the reference ACME type I in USA300 (6, 7) or based on PCR amplification and subsequent sequence analysis of ACME-*arc* and -*opp3* genes (2, 3, 5, 8). Comprehensive characterization of ACME III could yield useful information regarding important features of ACME and its conservation, evolution and spread, such as into the epidemic methicillin-resistant S. aureus strain USA300.

We detected ACME III in 9/142 (6.3%) oral methicillin-susceptible S. epidermidis isolates from separate patient groups who (i) were orally healthy, (ii) had dental implants, or (iii) had periodontal disease, using PCR primers directed toward ACME-*arcA* (6) and ACME-*opp3* (ACME-opp3B_F, 5′-GGATTCGCCCAAGTGATGACC-3′ and ACME-opp3B_R, 5′-GACTGCTGGGTATGACGT-3′), using the USA300 strain M05/0060 (9), which harbors both the ACME-*arc* and *opp3* operons, as a positive PCR control. We did not detect ACME III in any of the 54 S. aureus isolates investigated from the same three patient groups. The genetic structure of three of these ACME IIIs harbored by S. epidermidis isolates recovered by oral rinse sampling of three separate patients (one with periodontal disease [P16OR1], one healthy patient [204OR1], and one healthy patient with a dental implant [I11OR1]) were characterized in detail using WGS. To our knowledge, this is the first comprehensive description of the structural organization of ACME III. Isolates were first sequenced using a MiSeq sequencer (Illumina, Essex, United Kingdom) with genomic DNA extraction and library construction performed as previously described (10). Reads were checked for quality, trimmed, and contigs were generated by *de novo* assembly using SPAdes version 3.6 (http://cab.spbu.ru/software/spades/). For each isolate subjected to MiSeq-based WGS, ACME-associated genes were identified on four different contigs. As the genes in these contigs differed considerably in composition and orientation to those previously described in ACME types I and II and an appropriate reference ACME to use as a sequence scaffold was lacking, these isolates were also sequenced using a Pacific Biosciences (PacBio) RS sequencing system (CA, USA) with subsequent hierarchal genome assembly process (HGAP.3) analysis (The Genome Analysis Centre [TGAC], Norwich, United Kingdom) at an average coverage of 265×. For each isolate, all ACME-associated genes were identified on the same contig, thus confirming the orientation and synteny of all ACME III-associated genes.

The bioinformatic tools used for annotation and analysis were the BioNumerics Genome Analysis Tool (GAT) plug-in version 7.6 (Applied Maths, Sint-Martens-Latem, Belgium), Artemis sequence viewer (11), Artemis Comparison Tool (12) and BLAST software (https://blast.ncbi.nlm.nih.gov/Blast.cgi). Final elucidated genomic structures were confirmed using specific PCR primers (Table S1). The multilocus sequence types (MLST) of all three isolates investigated were determined by submitting the relevant genomic regions to the S. epidermidis MLST online database (https://pubmlst.org/sepidermidis/).

**FIG 1:**
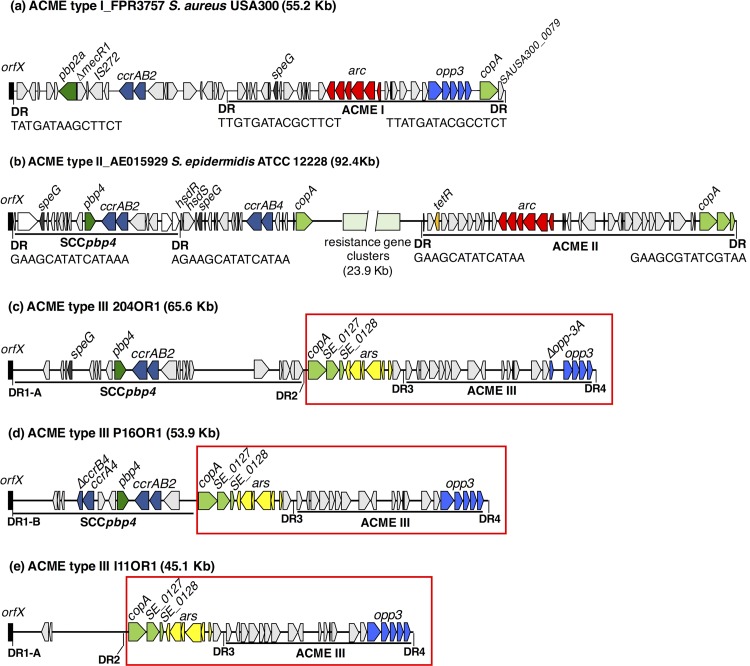
Schematic diagram showing the genetic organization of previously described ACME type I (a) and II (b) elements and the comparative organization of the three ACME III elements (c-e) determined by whole-genome sequencing in the present investigation. Arrows indicate the position and orientation of open reading frames. Genes commonly associated with antimicrobial resistance, SCC, or ACME are shaded in color; ACME-*arc* (red), *opp3* (blue), *speG* (dark gray), *copA* (lime green), *ars* operon (yellow), *pbp* (dark green), *ccr* (navy) and *tetR* (mustard). The resistance gene clusters encoding mercury and cadmium resistance in ACME type II_AE015929 are indicated in pale green. For each ACME, *orfX* is indicated in black and specific direct repeat sequences (DRs) identified are indicated (DR1-A, GAAGCGTATCACAAATAA; DR1-B, GAAGCATATCATAAGTGA; DR2, GAAGGGTATCATAAATAA; DR3, GAAGCGTATAATAAGTAA; DR4 GAAGCGTATCGTAAGTGA). Genomic regions from *copA* to DR4 in each ACME III exhibited >99% DNA sequence homology to each other and are enclosed in red rectangles.

Each ACME III harbored the *opp3* genes but lacked the *arc* operon and ranged from 21.2 to 21.5 kb in size. Adjacent staphylococcal cassette chromosome (SCC) elements were identified upstream of ACME III in two isolates (Fig. 1). Five distinct direct repeat sequences (DRs) (1-A, 1-B, and 2-4) were identified among the ACMEs characterized. Four (DR1-A and DR2-4; Fig. 1) were identified in the ACMEs harbored by isolates 204OR1 and I11OR1, whereas three (DR1-B, DR3, and DR4) were detected in the isolate P16OR1ACME (Fig. 1). There were four nucleotide differences identified between DR1-A and DR1-B (Fig. 1).

A comparative BLAST analysis of the DNA sequence for ACME III (the region between DRs 3 and 4 of isolate I11OR1) with ACME types I and II revealed that although the DNA sequence identity with ACME I (GenBank accession number FPR3757) and ACME II (GenBank accession number AE015929) was 99% and 96%, respectively, the query cover was only 54% and 60%, respectively, indicating high genetic similarity in distinct genomic regions only. These findings were confirmed using the Artemis Comparison Tool.

The *copA* gene and the *ars* operon were located directly upstream of ACME III for the first time. Previous studies described their location near the 3′ end and immediately downstream of ACME types I and II (1). In two of the elements sequenced (204OR1 and I11OR1), the *copA* and *ars* genes were located between DRs 2 and 3, whereas in the third ACME these genes were in the same location but DR2 was absent (Fig. 1). The genomic regions from the *copA* gene to DR4 exhibited >99% DNA sequence identity in all three ACMEs characterized. The relocation of these antimicrobial resistance genes has not been reported previously, although other genes encoding tetracycline, cadmium, mercury, and beta-lactam resistance have been detected previously within ACME-SCC composite elements (1).

Genes previously associated with the SCC*pbp4*-ACME II composite element in S. epidermidis (1) were identified in two isolates investigated (Fig. 1), including the cassette chromosome recombinase (*ccr*) and *pbp4* genes. Together, these findings highlight the ability of ACMEs to accumulate antimicrobial resistance genes, particularly within composite elements, and their potential to facilitate the spread of these genes to different strains and species.

The *speG* gene conferring polyamine resistance was identified in only one ACME III sequenced and previous research has suggested an association of this gene with *arcA*, which is absent in ACME III (13).

The main feature of ACME III is considered to be the presence of the *opp3* operon in the absence of the *arc* operon. The function of ACME-*opp3* has not been fully elucidated to date, but multiple different *opp* operons have been identified in bacterial species and are reportedly involved in nutrient uptake, host cell attachment, cell wall metabolism, resistance to antimicrobial peptides, and chemotaxis (11, 12). This operon was detected 510 bp upstream of DR4 in all three ACMEs characterized; however, a nucleotide deletion identified at the +384 position of the *opp3A* gene in isolate 204OR1 resulted in a frameshift mutation and the premature truncation of the encoded protein. These ACME-*opp3* genes likely contribute little advantage, perhaps due to the presence of two native *opp* operons in staphylococci, and perhaps represent remnants from previous ACME rearrangements.

The elements characterized were divided into modular segments by DRs (Fig. 1) in which the genomic regions between the *copA* gene and DR4 were highly conserved. Only eight of the 20 open reading frames observed in ACME III shared >97% sequence homology with the *opp3* operon and surrounding genomic regions of previously described ACME I (1); however, the *copA* and SE_0128 genes (corresponding to *copA* and SAUSA300_0079 in FPR3757) at the 3′ end of ACME I have been internalized in these ACME III-SCC composite elements (Fig. 1). Previous research has suggested a stepwise assembly of modular ACME segments in S. epidermidis prior to transfer to USA300 (14). The results of the present study support this hypothesis, demonstrate how mobile genetic elements can be constructed in a stepwise manner at this genomic region, and suggest that ACME III is most likely a genetic remnant of these processes. Surprisingly, all three isolates were identified as belonging to multilocus sequence type ST329. Previous MLST-based studies from this laboratory (unpublished) that investigated 36 independent oral S. epidermidis isolates identified 18 distinct STs, not including ST329. ST329 has been identified in only 3/1068 (0.3%) allelic profiles currently listed in the S. epidermidis MLST database (accessed 8 June 2017), suggesting that this ST is rare and is possibly the ST in which ACME rearrangements resulting in ACME type III originally occurred.

### Accession number(s).

The nucleotide sequences of the three ACME-SCC composite elements 204OR1, P16OR1, and I11OR1 have been submitted to GenBank under accession numbers MF346683, MF346684, and MF346685, respectively.

## Supplementary Material

Supplemental material
