# Supplementary material for: First Detailed Genetic Characterization of the Structural Organization of Type III Arginine Catabolic Mobile Elements Harbored by Staphylococcus epidermidis by Using Whole-Genome Sequencing
Source: Antimicrob Agents Chemother. 2017 Sep 22;61(10):e01216-17. doi: 10.1128/AAC.01216-17 (PMC5610516; doi:10.1128/AAC.01216-17)
Supplement: Supplemental material [file supp_61_10_e01216-17__index.html]

Supplemental material 

# First Detailed Genetic Characterization of the Structural Organization of Type III Arginine Catabolic Mobile Elements Harbored by Staphylococcus epidermidis by Using Whole-Genome Sequencing

## Supplemental material

- Supplemental file 1 -

  Table S1

  PDF, 54K
