# Supplementary material for: First Detailed Genetic Characterization of the Structural Organization of Type III Arginine Catabolic Mobile Elements Harbored by Staphylococcus epidermidis by Using Whole-Genome Sequencing
Source: Antimicrob Agents Chemother. 2017 Sep 22;61(10):e01216-17. doi: 10.1128/AAC.01216-17 (PMC5610516; doi:10.1128/AAC.01216-17)
Supplement: Supplemental material [file AAC.01216-17_zac010176567s1.pdf]

Table S1. Primers used to confirm the structure of ACME III

Primer Name	Sequence (5′-3′)	Amplimer Size (bp)	Isolates from which amplimer was obtained
**204-1F**<br>**204-2R**	CCGTTAAGGATTCATAAGGC<br>GCAGTCCTGTTGTTACAGTTG	1481	204OR1
**204-3F**<br>**204-4R**	ATGCAGAAACGTTCAGAGA<br>CTTCTGACAGCTCTTCTATTCC	3892	204OR1
**204-5F**<br>**204-6R**	ATCTTTGGAACCTGGACA<br>CTGTTCTACTGGAGTATGTGGTC	4999	204OR1, P16OR1
**204-7F**<br>**204-8R**	TAGGTTCTCGTGCCATTG<br>CTCATTACGGTCGCTTAGT	2929	204OR1, P16OR1, I11OR1
**204-9F**<br>**204-10R**	AGATGATGAGATGGCACG<br>CTAAAGCCGTATCCTAAGTTG	2444	204OR1, P16OR1, I11OR1
**I11-1 F**<br>**I11-2 R**	GGTAAATACGTAATATCGGTTG<br>GGGTGCGAGATGAATTAC	2493	I11OR1
**I11-3 F**<br>**I11-4 R**	CCACACACTTTAGCAGAATC<br>CTCTTATCGCCACTGATG	2943	I11OR1
**I11-5F**<br>**I11-6R**	GCTTGCTTAAAAATTGAGG<br>CCTGAGTGAAATTATTGACG	2027	204OR1, I11OR1
**P16-1F**<br>**P16-2R**	GTCCACCTTTTTATTAATAGGG<br>GGTCTTTTAGTTGATTCAATTC	2362	P16OR1
**P16-3F**<br>**P16-4R**	GATGGAAGTCACAGTATTCTTTG<br>CTTTTATCGCCACTGATGG	5998	P16OR1
